# Single-port thoracoscopic anatomic resection for chronic inflammatory lung disease

**DOI:** 10.1186/s12893-021-01252-w

**Published:** 2021-05-18

**Authors:** Chen-Yu Wu, Ying-Yuan Chen, Chao-Chun Chang, Yi-Ting Yen, Wu-Wei Lai, Wei-Li Huang, Yau-Lin Tseng

**Affiliations:** 1 Division of Thoracic Surgery, Department of Surgery, National Cheng Kung University Hospital, College of Medicine, National Cheng Chung University, 138 Sheng-Li Road, Tainan, 704 Taiwan; 2 Institute of Clinical Medicine, College of Medicine, National Cheng Kung University, Tainan, Taiwan

**Keywords:** Anatomic resection, Bronchiectasis, Inflammatory lung disease, Mycobacterial infection, Single-port, Video-assisted thoracoscopic surgery

## Abstract

**Background:**

It is challenging to proceed thoracoscopic anatomic resection when encountering severe pleural adhesion or calcified peribronchial lymphadenopathy. Compared with multiple-port video-assisted thoracoscopic surgery (MP-VATS), how to overcome these challenges in single-port (SP-) VATS is still an intractable problem. In the present study, we reported the surgical results of chronic inflammatory lung disease and shared some useful SP-VATS techniques.

**Methods:**

We retrospectively assessed the surgical results of chronic inflammatory lung disease, primarily bronchiectasis, and mycobacterial infection, at our institution between 2010 and 2018. The patients who underwent SP-VATS anatomic resection were compared with those who underwent MP-VATS procedures. We analyzed the baseline characteristics, perioperative data, and postoperative outcomes, and illustrated four special techniques depending on the situation: flexible hook electrocautery, hilum-first technique, application of Satinsky vascular clamp, and staged closure of bronchial stump method.

**Results:**

We classified 170 consecutive patients undergoing thoracoscopic anatomic resection into SP and MP groups, which had significant between-group differences in operation time and overall complication rate (*P* = 0.037 and 0.018, respectively). Compared to the MP-VATS group, the operation time of SP-VATS was shorter, and the conversion rate of SP-VATS was relatively lower (3.1% vs. 10.5%, *P* = 0.135). The most common complication was prolonged air leakage (SP-VATS, 10.8%; MP-VATS, 2.9%, *P* = 0.045).

**Conclusions:**

For chronic inflammatory lung disease, certain surgical techniques render SP-VATS anatomic resection feasible and safe with a lower conversion rate.

## Introduction

Minimally invasive surgery has become known worldwide to treat cancer because of the advantages of smaller surgical wounds, such as less acute pain and chronic paresthesia [1, 2]. However, in cases of long-term lung inflammation, severe hypervascularized pleural adhesion and calcified peribronchial lymph nodes (LNs) develop, making it challenging to proceed with thoracoscopic anatomic resection. Therefore, many surgeons prefer thoracotomy than minimally invasive surgery for chronic inflammatory lung diseases [3].

With the advancement of instruments and surgical techniques, video-assisted thoracoscopic surgery (VATS) is also used to treat symptomatic inflammatory lung disease, improving quality of life. The use of multiple-port (MP-) VATS for bronchiectasis, aspergillosis, and tuberculosis, has been reported previously [3–5]. We also published our results, showing the feasibility of MP-VATS lung resection for pulmonary tuberculosis [6–8]. Furthermore, we reported the outcome of single-port (SP-) VATS on pulmonary tuberculosis and sequestration despite the small number of cases [8, 9]. Compared to MP-VATS, the working space for instruments is so limited in SP-VATS, impeding the management of pleural adhesion, fissure symphysis, and calcified peribronchial LNs. Therefore, some special techniques should be applied to prevent unexpected thoracotomy. With more experiences in the past years, we had developed several useful techniques to overcome these difficulties. In the current study focused on chronic inflammatory lung disease, primarily bronchiectasis and mycobacterial infection, we shared the surgical outcomes of SP-VATS anatomic lung resection, compared to MP-VATS, and emphasized the importance of these techniques in preventing thoracotomy conversion and intraoperative complications.

## Patients and methods

### Patients and data collection

We reviewed all patients who underwent VATS anatomic resection for pulmonary mycobacterial infection or symptomatic bronchiectasis between January 2010 and December 2018 at the National Cheng Kung University Hospital using data from the database of thoracic surgery and medical records. If the chronic lung disease is characterized by chronic cough, recurrent pulmonary infection, life-threatening hemoptysis, and localized lung parenchyma change on computed tomography, we advise surgical resection to control infection and improve the quality of life. Besides, some patients of mycobacterial infection were enrolled as surgical candidates when cavitated lesions or destructed lung become bacterial refuges. Over the last few years of study period, SP-VATS has replaced MP-VATS and become the standard manner of surgical approach. However, when the preoperative computed tomography revealed the central hilar area is significantly involved by these lesions, we absolutely choose the intentional open thoracotomy because hand-assisted hemostasis is usually required in these situations. We excluded those with simultaneous lung abscess and empyema because of acute infection entity. This study, which did not require an informed consent, was approved by the National Cheng Kung University Hospital Institutional Review Board (approval no. B-ER-107-108).

We classified patients into two groups: SP-VATS and MP-VATS. The statistical analysis includes the following variables: (1) demographic and clinical information (i.e., sex, age, disease etiology, comorbidity, preoperative pulmonary function test, surgical indications, sidedness, extent of resection, and procedure type) and (2) the operation characteristics (i.e., the presence of calcified LNs, adhesion score, operation time, major vessel injury, the presence of conversion, chest tube duration, intensive care unit and hospital stay, and complications).

### Calcified LN and total adhesion score

Calcified LN is the calcification of LNs surrounding the bronchovascular bundle of the target lobe by the mediastinal window of the preoperative chest computed tomography (40 H level and 300 H width). When calcified LNs are present, the MP-VATS approach may be chosen intentionally depending on the surgeon’s preference.

The total adhesion score is the sum of the pleural adhesion score and fissure adhesion scores. The first intraoperative step was determining the individual severity of pleural and fissure adhesions subjectively as follows: 0 represents no or only focal adhesion; 1 limited adhesion around the target area with less than 50% of pleural cavity or interlobar area; and 2 diffuse adhesion or fused fissure with more than 50% of pleural cavity or interlobar area. Upon VATS exploration, we evaluated SP-VATS’ feasibility using the total adhesion score. If the score is ≧ 3 points, the surgeon could change to MP-VATS based on his judgement.

### Surgical procedure

All patients were intubated with a double-lumen endotracheal tube by an experienced anesthesiologist and placed in the lateral decubitus position. A single incision, measuring approximately 4 cm in length, was made in the fourth or fifth intercostal space at the anterior axillary line. The surgical techniques and instruments used in SP-VATS were similar to those used in MP-VATS. If unexpected situations during VATS require conversion, minithoracotomy will be performed. Typically, we divided the pulmonary arteries first to avoid congestion of the targeted lobe. The pulmonary veins and the bronchus were then dissected separately. The bronchus was divided using a linear stapler (Echelon Flex Endocutter, Johnson & Johnson, USA), but bronchial stump reinforcement was not routinely performed. After meticulous air leakage control, we placed a 24 Fr chest tube.

### Special techniques

**Fig. 1:**
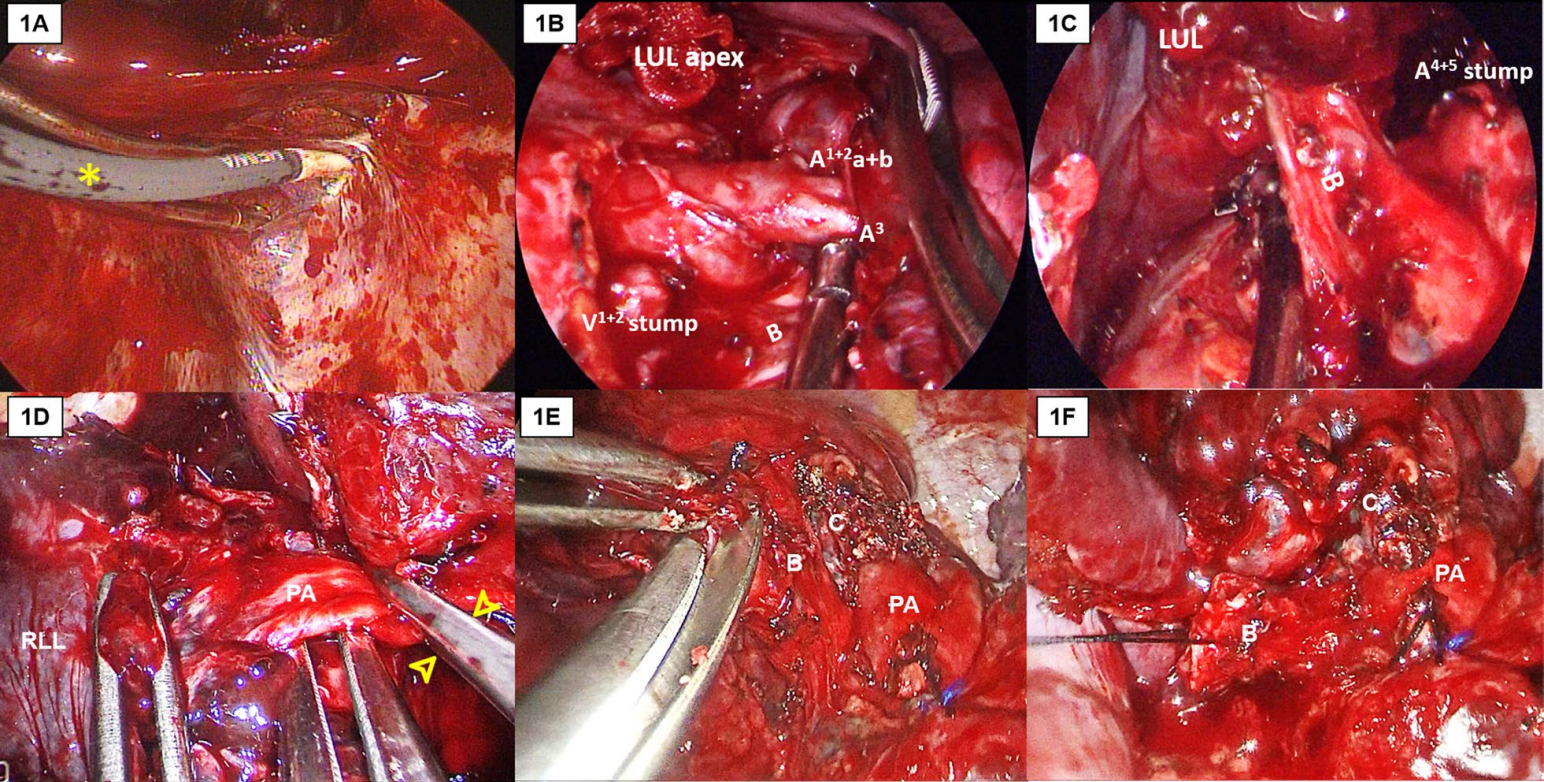
Special techniques for SP-VATS anatomic resection in chronic inflammatory lung disease. The first method is the use of a flexible hook electrocautery for apical or costophrenic adhesion (**A**, asterisk). The second method is the hilum-first technique, which can be used when the hilum area is relatively less adhesive than the apical area in mycobacterial infection. The LUL apex was still adhesive to the chest wall during hilar dissection (**B**). All vascular branches to the LUL were divided, followed by LUL bronchus transection (**C**). The last step was the lysis of the apical adhesion. The third method is the use of a Satinsky vascular clamp for bleeding prevention or control (**D**, arrows). The fourth method is bronchial stump staged closure when calcified LNs hinder the separation of the bronchus and vascular structure (**E** and **F**). *B* bronchus, *C* calcified lymph node, *LUL* left upper lobe, *PA* pulmonary artery, *RLL* right lower lobe

Because the limitations of SP-VATS, such as being a single-direction approach and using few instruments in the utility wound, we developed certain useful techniques to facilitate anatomic resection (Fig. 1).

First is the flexible hook electrocautery (Fig. 1A). Generally, blunt dissection using fingers or small peanut gauzes managed dense pleural adhesions. L-hook electrocautery and energy-based instruments were used to accelerate adhesiolysis. However, it is hard to use linear instruments for pleural adhesion in the apical or costophrenic area. So, we used flexible hook electrocautery to complete adhesiolysis.

The second technique is the hilum-first approach. After VATS exploration and adhesion severity evaluation, we could begin with the dissection of vascular structure if hilar adhesion was relatively minor rather than a dense pleural adhesion (Fig. 1B, C). Pleural adhesion could be used as counter traction, and we manipulate a single energy device and a suction device to avoid instrument fencing.

The third technique is the use of Satinsky vascular clamp (Fig. 1D). When faced with the occurrence of major bleeding or fear of main pulmonary artery injury during dissection, we apply a Satinsky vascular clamp immediately via the utility wound to clamp the vessel and bronchus concomitantly to control or prevent bleeding.

The final technique is the staged closure of the bronchial stump method (Fig. 1E). We applied this technique when segmental pulmonary artery is behind the bronchus and unable to be skeletonized due to calcified LNs. Using endoscopic scissors, the bronchus can be transected and the fibrocalcified tissue can be dissected sequentially along the posterior wall of the opened bronchial stump step by step. Then, the segmental pulmonary arteries can be looped and divided sequentially using either suture ligation or stapler device. Finally, the opened bronchial stump can be lifted by stay sutures and closed using a linear stapler (Fig. 1F).

### Statistical analysis

Descriptive statistics were used to assess the patients’ demographic and perioperative characteristics. Continuous data were expressed as means ± standard deviation, whereas categorical data were expressed as frequencies and proportions. Continuous variables were analyzed using the Mann–Whitney test, and categorical variables using the chi-squared test. A probability value of less than 0.05 was considered statistically significant. All statistical analyses were performed using IBM SPSS Statistics for Windows, version 19. (IBM Corp., Armonk, N.Y).

## Results

**Fig. 2:**
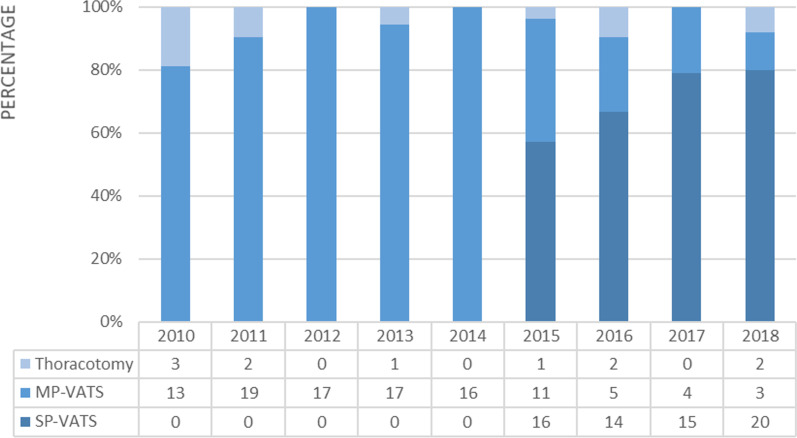
VATS anatomic resection for chronic inflammatory lung disease, primarily bronchiectasis, and mycobacterial infection, at the National Cheng Kung University Hospital. The patients intentionally undergoing open thoracotomy are also listed

Between January 2010 and December 2018, 181 patients underwent anatomic resection for bronchiectasis or pulmonary mycobacterial infection in our hospital. We performed VATS intentionally for 170 patients and open thoracotomy for 11 patients. Figure 2 shows the distribution of surgical approach. We began using SP-VATS in 2015 and generally adapted this method for lung surgery rapidly. By 2018, we used SP-VATS for approximately 87% of patients with chronic inflammatory lung disease. Only three patients underwent MP-VATS, one of whom had severe pleural and fissure adhesion (adhesion score 4) and two based on the surgeon’s preference (both adhesion score 0).

Table 1 lists the characteristics of patients who underwent VATS anatomic resection, with no significant between-group difference on age, sex, etiology, pulmonary function test, and surgical indication. The percentage of pulmonary mycobacterial infection in MP-VATS was slightly higher than that in SP-VATS (60% vs. 52.3%; *P* = 0.325). A total of 72 (42.4%) patients had comorbidities, including 21 (32.3%) who underwent SP-VATS and 51 (48.6%) MP-VATS. The prevalence of diabetes mellitus was significantly higher in the MP-VATS group than in the SP-VATS (32.4% vs. 18.5%; *P* = 0.047).

**Table 1:** Patient characteristics by surgical approach

	SP-VATS (n = 65)	MP-VATS (n = 105)	*P-*value
Age, mean ± SD	54.2 ± 12.6	52.4 ± 12.5	0.351
Sex, male (%)	33 (50.8)	56 (53.3)	0.745
Etiology, n (%)			0.325
Bronchiectasis	31 (47.7)	42 (40.0)	
TB/NTM infection	34 (52.3)	63 (60.0)	
Comorbidity, n (%)			
Hypertension	12 (18.5)	18 (17.1)	0.827
Diabetes mellitus	12 (18.5)	34 (32.4)	0.047
Coronary artery disease	1 (1.5)	3 (2.9)	1.000
GERD	0	3 (2.9)	0.287
CKD/ESRD	1 (1.5)	3 (2.9)	1.000
Liver cirrhosis	1 (1.5)	3 (2.9)	1.000
Malignancy history	3 (4.6)	2 (1.9)	0.372
Pulmonary function test, mean ± SD			
FEV1 (L)	2.16 ± 0.84	2.27 ± 0.80	0.458
PEF1/FVC (%)	76.9 ± 11.0	77.5 ± 10.3	0.755
Surgical indication, n (%)			0.066
Hemoptysis	36 (55.4)	48 (45.7)	
Recurrent pneumonia	11 (16.9)	16 (15.2)	
Cavity, infected	11 (16.9)	13 (12.4)	
Destroyed lobe	2 (3.1)	4 (3.8)	
TB medication failure	3 (4.6)	23 (21.9)	
Undetermined mass	2 (3.1)	1 (1.0)	
Sidedness, n (%)			0.281
Right	35 (53.8)	55 (52.4)	
Left	27 (41.5)	49 (46.7)	
Bilateral	3 (4.6)	2 (1.0)	
Extent of resection, n (%)			0.435
Upper lobe	32 (49.2)	63 (60.0)	
Middle lobe	9 (13.8)	16 (15.2)	
Lower lobe	15 (23.1)	20 (19.0)	
Upper + middle lobe	3 (4.6)	2 (1.9)	
Upper + lower lobe	3 (4.6)	3 (2.9)	
Middle + lower lobe	3 (4.6)	1 (1.0)	
Surgical procedure, n (%)			0.226
Lobectomy	27 (41.5)	52 (49.5)	
Segmentectomy	28 (43.1)	47 (44.8)	
Lob. + Seg., ipsilateral	6 (9.2)	4 (3.8)	
Lob. + Seg., bilateral	1 (1.5)	1 (1.0)	
Seg. + Seg., ipsilateral	0	1 (1.0)	
Seg. + Seg., bilateral	2 (3.1)	0	
Bilobectomy	1 (1.5)	0	

*CKD/ESRD* chronic kidney disease/end-stage renal disease, *FEV1* forced expiratory volume in 1 s, *FVC* forced vital capacity, *GERD* gastroesophageal reflux disease, *Lob.* Lobectomy, *NTM* nontuberculous mycobacteria, *SD* standard deviation, *Seg.* Segmentectomy, *TB* tuberculosis

The sidedness and extent of resection, as well as surgical procedure, were also similar between the two groups (Table 1). A total of 181 anatomic resection procedures were performed, including 88 lobectomies and 93 segmentectomies. Among these patients, four underwent simultaneous bilateral anatomic resection (three for bronchiectasis and one for nontuberculous mycobacterial infection).

Table 2 summarizes the surgical characteristics of the two groups. No significant between-group difference was observed on calcified LN, the severity of pleural /fissure adhesion, and adhesion score. Although the percentage of high adhesion score (score = 4) in the MP-VATS group was higher than that in the SP-VATS group, the comparison was not statistically significant (score 0–3 vs. score 4; *P* = 0.113). No significant difference was also observed on intraoperative blood loss, major vessel injury, conversion rate, chest tube duration, intensive care unit stay, or postoperative hospital stay. SP-VATS patients had less operation time but a higher complication rate than MP-VATS patients (*P* = 0.037 and 0.018, respectively). Two (3.1%) patients were converted to mini-thoracotomy for bleeding control, both of which were in the early period of SP-VATS development. There was no 30-day mortality in either group.

**Table 2:** Perioperative data of SP- and MP-VATS anatomic resection for chronic inflammatory lung disease

	SP-VATS (n = 65)	MP-VATS (n = 105)	*P-*value
Calcified LNs, n (%)	8 (12.3)	20 (19.0)	0.250
Pleural adhesion (A), n (%)			0.269
No or focal (0)	44 (67.7)	58 (55.2)	
Limited, < 50% of pleural cavity (1)	9 (13.8)	19 (18.1)	
Diffuse, ≧50% of pleural cavity (2)	12 (18.5)	28 (26.7)	
Fissure adhesion (B), n (%)			0.226
No or focal (0)	33 (50.8)	57 (54.3)	
Limited, < 50% of interlobar area (1)	18 (27.7)	18 (17.1)	
Diffuse or fused, ≧50% of interlobar area (2)	14 (21.5)	30 (28.6)	
Adhesion score (A + B), n (%)			0.221
0	24 (36.9)	43 (41.0)	
1	20 (30.8)	18 (17.1)	
2	9 (13.8)	14 (13.3)	
3	6 (9.2)	11 (10.5)	
4	6 (9.2)	19 (18.1)	
Adhesion score, binary grading, n (%)			0.113
Low (Score 0–3)	59 (90.8)	86 (81.9)	
High (Score 4)	6 (9.2)	19 (18.1)	
Operation time (min), mean ± SD	186.3 ± 67.8	211.6 ± 79.7	0.037
Intraoperative blood loss (mL), mean ± SD	256.2 ± 412.1	300.0 ± 431.4	0.515
Major vessel injury, n (%)^a^	9 (13.8)	6 (5.8)	0.076
Conversion, n (%)	2 (3.1)	11 (10.5)	0.135
Chest tube duration (days)			0.184
Mean ± SD	6.7 ± 15.4	4.1 ± 3.4	
Median (IQR)	3.0 (3.3)	3.0 (1.0)	
ICU stay (days)			0.407
Mean ± SD	0.7 ± 1.6	0.6 ± 1.2	
Median (IQR)	0 (1.0)	0 (1.0)	
Postoperative hospital stay (days)			0.107
Mean ± SD	8.4 ± 10.5	6.2 ± 3.6	
Median (IQR)	5.0 (4.0)	5.0 (3.0)	
Complication, n (%)	19 (29.2)	15 (14.3)	0.018

*ICU* intensive care unit

^a^Major vessel injury is the injury of pulmonary artery, and suture repair is needed

Table 3 lists the details regarding the complications. On the whole, 34 (20%) patients experienced complications and 26 (15.3%) had pulmonary-related complications. The most common complication was prolonged air leakage (> 7 days), followed by post-surgical empyema (5.9% and 4.1%). The SP-VATS group had a significantly higher percentage of prolonged air leakage (10.8% vs. 2.9%, *P* = 0.045).

**Table 3:** Complications of SP-VATS vs. MP-VATS anatomic resection for chronic inflammatory lung disease

	SP-VATS	MP-VATS	*P*-value
Prolonged air leak > 7 days, n (%)	7 (10.8)	3 (2.9)	0.045
Post-surgical empyema, n (%)	5 (7.7)	2 (1.9)	0.108
Massive hemothorax, n (%)	1 (1.5)	0	0.382
Massive hemoptysis, n (%)	0	2 (1.9)	0.525
Pneumonia, n (%)	1 (1.5)	2 (1.9)	1.000
Respiratory failure with tracheostomy, n (%)	1 (1.5)	0	0.382
Wound infection, n (%)	1 (1.5)	2 (1.9)	1.000
Arrhythmia, n (%)	2 (3.1)	3 (2.9)	1.000
AUR, n (%)	1 (1.5)	1 (1.0)	1.000
Others^a^, n (%)	2 (3.1)	0	0.145

*AUR* acute urinary retention

^a^Others included one case of neutropenic fever and another of cerebrovascular accident

Among the 65 consecutive patients of SP-VATS, patients 1–33 constitute the first half phase and patient 34–65 constituted the second half phase. Table 4 demonstrated the complication rate regarding the different phase. No significant between-group differences were observed in each complication, but the overall complication rate showed a decreasing trend in the second half phase.

**Table 4:** The complication of SP-VATS anatomic resection for chronic inflammatory lung disease regarding the different phase

	First half phase n = 33	Second half phase n = 32	*P*-value
Overall, n (%)	12 (36.4)	7 (21.9)	0.202
Prolonged air leak > 7 days, n (%)	4 (12.1)	3 (9.4)	0.723
Post-surgical empyema, n (%)	3 (9.1)	2 (6.2)	0.670
Massive hemothorax, n (%)	1 (3.0)	0	0.325
Pneumonia, n (%)	1 (3.0)	0	0.325
Respiratory failure with tracheostomy, n (%)	1 (3.0)	0	0.325
Wound infection, n (%)	0	1 (3.1)	0.310
Arrhythmia, n (%)	1 (3.0)	1 (3.1)	0.982
AUR, n (%)	1 (3.0)	0	0.325
Others^a^, n (%)	2 (6.1)	0	0.160

*AUR* acute urinary retention

^a^Others included one case of neutropenic fever and another of cerebrovascular accident

### Comment

Dense pleural adhesion is a relative contraindication of VATS [4]. With advancements in thoracoscopic video systems and surgical techniques, VATS anatomic resection can be applied to chronic inflammatory lungs even with pleural adhesions, including pulmonary mycobacterial infection, bronchiectasis, or lung cancer after neoadjuvant treatment. Currently, pleural or fissure adhesions do not preclude proceeding with MP-VATS [10]. However, literature focused on how to overcome dense adhesion using SP-VATS is still limited. We reported our experiences on dealing with pulmonary tuberculosis by thoracotomy and MP-VATS previously [9]. With well-developed VATS skills, we began performing SP-VATS anatomic resection in 2015, the results of which were reported in the current study. On the basis of these experiences, we established several strategies to overcome some of this technique’s difficulties.

First is using appropriate instrument for pleural adhesiolysis. In SP-VATS, it is relatively easy to release mediastinal and mid-thoracic pleural adhesion by electrocautery. However, for the adhesion of the lung apex, particularly the anterior apical area, bended electrode, or straight L-hook electrocautery could barely reach it. To overcome this limitation, a flexible L-hook electrocautery is used to release pleural adhesion in these tricky areas. Mun et al. also described a similar experience with the management of pleural adhesion via three- or four-port VATS [11]. Using suitable instruments for adhesiolysis, lung parenchyma injury, which leads to intraoperative bleeding or postoperative air leakage, can also be decreased.

Second is the management strategies of pleural and fissure adhesion. In SP-VATS, the most important is the single-direction manner, either anterior-to-posterior or caudal-to-cranial approach [12]. In our experience, around one-fourth of the patients with chronic inflammatory disease wound have severely adhesive or fused fissure, which resulted in higher risk of vessel injury during fissure opening. Fissure-last single-direction approach is better than fissure-first approach in decreasing the difficulty of vascular skeletonization and reducing the possibility of conversion and also decreasing the incidence of postoperative prolonged air leakage as the fissure is divided at the last step using staplers [13].

As facing apical adhesion usually caused by tuberculosis, the hilum-first technique, which we called apical adhesion-last approach, is an effective method. We had observed that some patients with chronic inflammatory lung disease, especially pulmonary tuberculosis, have intense apical pleural adhesion but relatively minor hilar adhesion. Dissecting this adhesion as the last step has several advantages. On the one hand, after transection of lobar arteries is done, oozing from lung parenchyma will decrease during pleura adhesiolysis. By contrast, it is difficult to control oozing from either neovascularized pleura or lung parenchyma if pleura adhesiolysis is done first. On the other hand, being separated from the chest wall, the poorly compliant upper lobe may obscure the surgical field and make hilar dissection difficult. For SP-VATS, it is definitively important to use fewer instruments to do more work simultaneously, such as suction, dissection, and traction. The adhesion on the roof can provide counter traction when approaching the hilum structure. This method is specifically beneficial for left upper lobe with chronic inflammatory disease resection.

The third method is concomitant clamping of the bronchovascular structures with temporary use of a Satinsky vascular clamp, which is feasible for lower or middle lobe resection if calcified LNs severely anchored to both the bronchus and lobar arteries. The simultaneous transection of the bronchus and the artery (simultaneously stapled lobectomy) has been reported [14–16]. When facing the fibrocalcified LN in bronchovascular buddle, however, we did not completely support this technique based on past experience of staple malformation and disruption, tissue tear, and massive bleeding from artery stump. In addition, calcified LNs in the bronchovascular bundle also contribute to vascular injury when attempting to skeletonize vessels. Gonzalez-Rivas et al*.* shared their experience with bleeding control using SP-VATS [17]. However, we cannot apply these methods if there is not enough space between the bronchus and vessels. Using this technique, we seldom need to convert to open thoracotomy hastily when artery injury occurs but rather can repair the injured site calmly. Dividing all the tissues surrounding the bronchovascular bundles, which facilitate the application of the Satinsky clamp, is the point of this method.

The last method is staged closure of the bronchus, which we have described previously [18]. In contrast to the third method used for the lower and middle lobe, it is rather suitable for bilateral upper lobe resection. Some surgeons have also adapted this technique to overcome the adherence of calcified LNs to the bronchus and lobar arteries [19]. The key point is that the bronchus should be transected by scissors as distally as possible to preserve a longer stump, facilitating its closure with a linear stapler sequentially.

From MP- to SP-VATS approach, we have developed several useful techniques to facilitate VATS anatomic resection for chronic inflammatory lung disease [9, 18]. Although the current study demonstrated that the incidence of intraoperative major vessel injury was still higher, the rate of conversion to thoracotomy was relatively lower in the SP-VATS group, and the between-group difference of intraoperative blood loss was minimal. This represents that we can manage these vascular injuries well by SP-VATS techniques. Noticeably, the operation time is significantly shorter in the SP-VATS group. We believed these are a result of the accumulation of VATS experience and proficiency of surgical techniques we provided. When it comes to postoperative recovery characteristics, because one patient with bronchiectasis had post-surgical empyema, resulting in prolonged chest tube drainage (116 days) and postoperative hospital stay (37 days), the average of chest tube drainage and postoperative hospital stay in SP-VATS is relatively higher than that in MP-VATS without statistical significance (p = 0.184 and 0.107, respectively). Based on the results of the median and IQR values, we considered SP-VATS to be a safe procedure as MP-VATS.

A higher complication rate with SP-VATS is unexpected, which is associated with insufficient experience in the developmental phase of SP-VATS. The primary complication was prolonged air leakage (> 7 days), which also resulted in a longer chest tube duration and longer hospital stay. The incidence of prolonged air leakage could be reduced when some prophylactic agents, such as fibrin glue or polyglycolic acid sheets (NEOVEIL), are applied, but they are not covered by our national health insurance [20]. Noticeably, there was no 30-day mortality in SP-VATS group although the complication rate is relatively higher.

This study had some limitations. First, this retrospective non-randomized study was conducted at a single institution, and the number of cases was small. Second, the severity of adhesion score is subjectively evaluated, and the long-term outcome data are limited. With increased experience in performing SP-VATS, the surgeon’s tolerance and perseverance for managing adhesion and calcified lymph node gradually increase, but surgical approach planning still depends on surgeon’s preference and disease complexity. In our view, minimally invasive surgery is a trend nowadays, and SP-VATS is widely discussed in lung cancer surgery. For chronic inflammatory lung disease, however, SP-VATS is still challenging. Therefore, we made a great effort to develop some techniques to facilitate SP-VATS anatomic resection. Moreover, these specifical techniques can also be applied in MP-VATS to reduce unexpected thoracotomy conversion.

In conclusion, pleural and fissure adhesion or calcified LNs around the vessels and bronchus are not obstacles for conducting SP-VATS. If the surgeon is acquainted with the techniques we have proposed, SP-VATS anatomic resection for chronic inflammatory lung disease could become more feasible and safer with a lower thoracotomy conversion rate.

## Data Availability

The datasets used and/or analyzed during the current study are available from the corresponding author on reasonable request.
